# Obtaining Self-Samples to Diagnose Curable Sexually Transmitted Infections: A Systematic Review of Patients’ Experiences

**DOI:** 10.1371/journal.pone.0124310

**Published:** 2015-04-24

**Authors:** Priyamvada Paudyal, Carrie Llewellyn, Jason Lau, Mohammad Mahmud, Helen Smith

**Affiliations:** 1 Division of Primary Care and Public Health, Brighton and Sussex Medical School, Brighton, United Kingdom; 2 North-West Thames London Deanery, London, United Kingdom; David Geffen School of Medicine at UCLA, UNITED STATES

## Abstract

**Background:**

Routine screening is key to sexually transmitted infection (STI) prevention and control. Previous studies suggest that clinic-based screening programmes capture only a small proportion of people with STIs. Self-sampling using non- or minimally invasive techniques may be beneficial for those reluctant to actively engage with conventional sampling methods. We systematically reviewed studies of patients’ experiences of obtaining self-samples to diagnose curable STIs.

**Methods:**

We conducted an electronic search of MEDLINE, EMBASE, CINAHL, PsychINFO, BNI, and Cochrane Database of Systematic Reviews to identify relevant articles published in English between January 1980 and March 2014. Studies were included if participants self-sampled for the diagnosis of a curable STI and had specifically sought participants’ opinions of their experience, acceptability, preferences, or willingness to self-sample.

**Results:**

The initial search yielded 558 references. Of these, 45 studies met the inclusion criteria. Thirty-six studies assessed patients’ acceptability and experiences of self-sampling. Pooled results from these studies shows that self-sampling is a highly acceptable method with 85% of patients reporting the method to be well received and acceptable. Twenty-eight studies reported on ease of self-sampling; the majority of patients (88%) in these studies found self-sampling an “easy” procedure. Self-sampling was favoured compared to clinician sampling, and home sampling was preferred to clinic-based sampling. Females and older participants were more accepting of self-sampling. Only a small minority of participants (13%) reported pain during self-sampling. Participants were willing to undergo self-sampling and recommend others. Privacy and safety were the most common concerns.

**Conclusion:**

Self-sampling for diagnostic testing is well accepted with the majority having a positive experience and willingness to use again. Standardization of self-sampling procedures and rigorous validation of outcome measurement will lead to better comparability across studies. Future studies need to conduct rigorous economic evaluations of self-sampling to inform policy development for the management of STI.

## Introduction

Sexually transmitted infections (STIs) impose an enormous burden on sexual and reproductive health worldwide. Over a million people acquire a STI every day and around 500 million new cases of curable STIs (chlamydia, gonorrhoea, syphilis and trichomoniasis) occur each year [1]. Despite surveillance, monitoring and multiple interventions aimed at prevention, diagnosis and treatment of STIs, these infections continue to place a significant burden on healthcare resources. In the UK, approximately 510,000 new STI diagnoses (other than HIV) were made in 2011, with estimated treatment costs of £620 million [2].

Routine screening is an important component of STI prevention and control. However, it has been recognised that an effective response to the global STI epidemic necessitates alternative strategies beyond the traditional approach of clinic-based sampling and conventional culture methods for screening [3] [4]. Earlier studies have reported that clinic based screening programmes capture only a small proportion of people with STIs; mainly due to barriers such as clinic inaccessibility, lack of privacy, embarrassment, discomfort, and the lack of time or financial resources needed to attend appointments [5] [6]. With the advent of new technologies in STI diagnosis, screening outside of the clinic environment is now more feasible than before. Self-sampling using non-invasive or minimally invasive sampling techniques such as urine samples, cervical-brush, vaginal swabs, and tampons have demonstrated an equivalent or superior detection of STIs compared to conventional sampling and detection methods [7]. These techniques have been found to be particularly beneficial for those where access to medical care is difficult [7] and for vulnerable individuals who are often reluctant to visit sexual health clinics due to fear of embarrassing and painful intimate examinations. Self-sampling is also helpful in screening for STIs where individuals affected may be completely asymptomatic, but if their condition is left undetected and untreated it could potentially lead to serious complications such as pelvic inflammatory disease (PID), infertility (as in the case of Chlamydia) or transmission to sexual partners [8]. Self-sampling is not only beneficial to the patient, but could be helpful in reducing healthcare costs by averting major complications of STIs [9] [10].

There has been a plethora of research on the diagnostic accuracy, sensitivity and specificity of self-sampling methods in relation to conventional ‘gold standards’. Fewer studies have looked at patients’ acceptance of these methods and preferences for self-sampling compared to conventional methods. Patient’s preferences and perspectives are important in successful tailoring of STI screening and treatment services. Earlier reviews have mostly focused on acceptability of HPV self-sampling [11,12] and HIV self-testing [13]. One recently published systematic review and meta-analysis compared the effectiveness and acceptability of home-based self-sampling and clinic based specimen collection for STIs [14]. However, the review only included a small number of studies and were confined to female participants. Our study aims to systematically review the literature of patients’ experiences, acceptability and preferences for self-sampling to diagnose curable STIs. We believe that the findings of this review will be helpful in formulating strategies to facilitate earlier diagnosis of STIs, which are acceptable to patients.

## Materials and Methods

This systematic review was conducted and reported following the PRISMA (Preferred Reporting Items for Systematic Reviews and Meta-Analyses) guidelines (http://www.prisma-statement.org/).

### Eligibility criteria

Studies were eligible if they (i) involved human participants who had undergone self-sampling for the diagnosis of curable STI(s) (ii) reported on patients’ experiences, acceptance, preferences, or willingness to undergo self-sampling (iii) included primary data; (iv) were published in English. Studies using any means of self-obtained samples including vaginal/rectal/penile/vulval swabs, cervical/vaginal lavage, cervical brush, tampons, urine, stools, throat gargle, or finger-prick blood spot for diagnostic purposes were included. Studies that were excluded were those addressing self-examination for cancer (such as breast/testicular examination), home-care, self-sampling for monitoring or management of pre-existing conditions (such as diabetes, blood pressure or anticoagulation therapy), acceptability of vaccinations or any form of home treatment. It was felt that these may reflect different views to self-sampling for diagnostic reasons. In addition, focus groups or other studies where the procedures were merely described and not experienced were not included.

### Information sources and Search

An electronic search was undertaken of the following databases to identify relevant articles published between January 1980 and March 2014: MEDLINE(R), EMBASE, CINAHL, PsychINFO, BNI, and Cochrane Database of Systematic Reviews. The bibliographies of included studies were also searched for relevant articles. Grey literatures, including conference abstracts, unpublished reports or other non-peer reviewed articles, were excluded from the search, as the quality of the contents cannot be assured.

### Search terms

Where advanced search engines were available, search terms for obtaining self-sample (self-sampl*, home-sampl*, self-test*, home-test*, self-swab*, home-swab*, self-collect*, home-collect*) were combined with search terms for patient opinion (Acceptab*, prefer*, experience*, feasib*). Where there were limited search options, the terms self-testing/sampling and acceptability, or preference, or experience or feasibility were used.

### Study selection, data extraction, and quality assessment

Two reviewers (PP and JL) independently carried out the search, screened the titles of the articles and removed any irrelevant and duplicate articles. Abstracts of the remaining articles were assessed applying the inclusion criteria. Any disagreements in selections were discussed and resolved during consensus meetings involving a third investigator (CL or HS).

The following relevant information were extracted from the selected studies: study characteristics (author, year of publication, country), disease, method of self-sampling, explanation of self-sampling procedure, location of sampling, total number of participants involved in study, total number of participants who provided opinion, response rate, method of ascertaining acceptability/experience (Table 1). The quality of reporting of individual studies was assessed by two independent reviewers (all authors involved) with reference to a standardised checklist of quality items recommended by the Strengthening the Reporting of Observational Studies in Epidemiology (STROBE) Statement [15].

**Table 1:** Summary of studies included in the systematic review.

Author, year, country	Disease	Method of self-sampling	Explanation of procedure/mode of instruction	Location of sampling	Type of patients	Sample (Opinion)	Response rate	Method of ascertaining acceptability/preferences
Markos 1994, UK [21]	Gonorrhoea, chlamydia	Cervical swab	Verbal with aid of plastic model	GUM clinic	F 18–50 yrs(median 24yrs)	75 (75)	NR	SAQ immediately after SS and CS
Macmillan 2000, UK [22]	Chlamydia	Urine, vulval swab	Verbal and illustrated written	Urban family planning clinic	F 14–25 yrs	103 (103)	68%	IAQ immediately after SS
Stephenson 2000, UK [23]	Chlamydia	Urine, vulval swab	Illustrated written	Home (Postal)Using data from 3 general practices	M 18–35 yrsF 18–25yrs(young people)	145 (NR) 27 F: swab38 F: urine80 M: urine	F 31 (39*)%M 36 (46*)%	SAQ (Postal)
Fenton 2001, UK [56]	Chlamydia	Urine	NR	Home Interviewers delivered and collected kit from home	M & F 18–44 yrs	143 (36)	72%F 73%M 65%	FID after SS on selected respondents (n = 36)
Wiesenfeld 2001, UK [47]	Chlamydia, gonorrhoea, trichomonas	Vaginal swabs	Illustrated written	School health centres	F 12–19 yrs(median 16yrs)(public high school students)	228 (228)	Approx75%	SAQ immediately after SS
Bloomfield2002, USA [54]	Chlamydia, gonorrhoea	Urine	Detailed written	Home/Community locations	M 16–67 yrs(median 43 yrs)(majority 95% white MSM)	80 (80)77 M 3 F	38%	SAQ (Postal)
Holland-Hall 2002, USA [24]	Chlamydia, gonorrhoea, trichomonas	Vaginal swab	Verbal and illustrated written	Juvenile correctional facility	F 12–17 yrs (adolescents)	133 (133)	85%	SAQ immediately after SS
Serlin 2002, USA [25]	Chlamydia, gonorrhoea, trichomonas	UrineVaginal swab	NR	Health clinicUniversity or health maintenance organization clinics	F 12–21 yrs(adolescents)	155 (155)	82%	IAQ immediately after SS and CS
Bloomfield 2003, USA [26]	Chlamydia	Urine	Detailed written instructions	Home (Postal)Kit offered to patients previously tested positive for chlamydia	M & F >18 yrs	63 (67)	18% (21%*)	SAQ (Postal)
Hsieh 2003, USA [48]	Chlamydia	Urine, vaginal swab	Illustrated written	Military Fort	F 17–36 yrs(Army recruits)	1403 (1382) urine+swab15 urine only6 swab only	31%	SAQ immediately after SS
Newman 2003, USA [27]	Chlamydia,gonorrhoea	Urine, vaginal swab	NR	Federal prison for women	F 18–52 yrs	614 (535) 596 urine+swab8 swab only10 urine only	82%	IAQ after SS (79 did not interview due to time constraints)
Pimenta 2003, USA [28]	Chlamydia	Urine	NR	77 GP practices and 29 secondary care clinics	F 16–24 yrs	16,930 (25)Acceptability only part of wider study	76%	FID (n = 25) arranged at a later date at venue of participant’s choice
Richardson 2003, Canada [16]	Chlamydia	Vaginal swab	Verbal and illustrated written	12 Family physicians’ office and 10 health care centres	F 16–30 yrs	514 (472)	NR (285 refusers)	SAQ immediately after SS
Tanksale 2003, India [42]	Bacterial vaginosis, candidiasis	Vaginal swab	Instructed and guided by gynaecologist	Gynaecological OPD of Goa Medical College	F 18–45 yrsattending OPD	75 (75)	NR	IAQ immediately after SS‘Preference was noted along with the reason’
Chandeying 2004, Thailand [60]	Chlamydia	Urine, vaginal swab, tampon	Clear instruction given by research staff	A Hospital gynaecological OPD	F age NR(523 commercial sex workers, 430 outpatient women)	953 (NR)570 All three 906 tampon +urine(Vaginal swab only second half of study)	94%	IAQ immediately after SSOnly one question: willingness to re-use SS in future
Tebb 2004, USA [58] (9 month follow-up of Serlin 2002 [25])	Chlamydia, gonorrhoea, trichomonas	Urine, vaginal swab	NR	Health clinicUniversity or health maintenance organization clinics	F 13–20 yrs(adolescents)	155 (98)	63%	TIAQ 9 months after initial SS
Chernesky 2005, USA [29]	Chlamydia, gonorrhoea	Urine, vaginal swab	Illustrated written	Eight study sites including family planning, STDs, teenagers, and student health clinics	F 15–71 yrs	1465 (1090)	Sampling NROpinion82%	SAQ immediately after SS and CS in 7/8 of the study sites
Gotz 2005, Netherlands [50]	Chlamydia	Urine	Written	Home (Postal)	M & F 15–29 yrs	754 (351)	50%	SAQ (Postal) sent 6–12 weeks after receiving result of chlamydia test
Gaydos 2006, USA [30]	Chlamydia	Vaginal swab	Illustrated written	HomeInternet/phone request or obtain in community	F 14–63 yrs (median 23 yrs)	400 (508)	NR	SAQ (Postal) received with samples; plus additional 108 internet-based questionnaires
Hoebe 2006, Netherlands [31]	Chlamydia, gonorrhoea	Urine, vaginal swab	Verbal aided by demonstration material	Public health clinic	F 16–35 yrs	413 (413)	100% Urine98.5% Vaginal swab	SAQ immediately after sampling
Van-de-Wijgert 2006 South Africa [32]	Chlamydia, gonorrhoea, bacterial vaginosis, candida species, HPV	Tampon, vaginal swab	Verbal	Community health centres	F 18–69 yrs	450 (450) 222 vaginal swabs228 tampon(300 non-STI; 150 with STI symptoms)	NR	IAQ immediately after SS and again during 2 weeks follow-up
Jones 2007, South Africa [33]	Chlamydia,gonorrhoea,trichomonas	Vaginal swab	Clinic group: NRHome group: Illustrated written	Recruited at 4 community-based youth groups and 2 public health clinics Randomised to SS at Home or SS at Clinic	F 14–25 yrs	278 (244) Home group: 146 (113)Clinic group: 132 (131)	45%47% Home group42% Clinic group	SAQ (Postal/at clinic site) immediately after SS; and IAQ at 6 weeks follow-up visit (for both home and clinic groups)
Lippman, 2007, Brazil [3]	Chlamydia, gonorrhoea, trichomonas	Vaginal swab	Illustrated written	Randomised to home or clinic self-sampling groups	F 18–40 yrs	787 (787)Home group: 393(393)Clinic group: 394(394)	86%	Questionnaire (Means of administration NR)
Papp 2007, USA [34]	Chlamydia, gonorrhoea	Oral-throat rinses gargle ~1/2 allocated to using mouth wash, the rest sterile water	Verbal Instructions	City based STD Clinic	M> 18 yrs Patients with indication for pharyngeal specimen collection	561 (561)561 chlamydia556gonorrhoea	48%	SAQ immediately after CS and SS
Mahilum Tapay 2007, UK [51]	Chlamydia	Vaginal swab,urine	Illustration instruction sheet	1 sexual health clinic and 2 GUM clinics	F 16–54 yrs attending sexual health centre and GUM clinics	1349 (1083)	80%	SAQ immediately after SS
Berwald 2009, USA [55]	Chlamydia, gonorrhoea	Vaginal swab	Written instruction	ED of a tertiary care centre and a municipal academic Level 1 trauma centre	F 18–55 yrsSexually active and visiting ED	162 (162)	56%	SAQ (open ended question documenting discomfort during sample collection)
Van der Helm 2009, Netherlands [35]	Chlamydia,gonorrhoea	Rectal swab	Verbal aided by illustrations	2 STI outpatient clinics	MSM & F 23–44 yrsReceptive anal intercourse in past 6 months	2394 (1845)MSM 1485 (1151)F 936 (694)	55%60% MSM48% F	SAQ immediately after SS and CS
Wayal 2009, UK [36] Wayal 2011, UK [57]	Chlamydia, gonorrhoea	Oral gargle, pharyngeal swab, rectal swab, mouth pads	Illustrated written	GUM Clinic	MSM >18 yrs	301 (274)	90%	SAQ immediately after SS; FID arranged for some participant (n = 24)
Brown 2010, UK [37]	Chlamydia, gonorrhoea	Urine, vaginal swab, throat swab (latter was not self-taken)	NR	GUM ClinicPatients randomly allocated to standard (n = 191) or self-sampling (n = 187)	M & F >16 yrs Attending clinic for first time or with new problem	187 (174)101 Urine93 vaginal swab	70%	SAQ immediately after clinic visit (mainly regarding clinic satisfaction but question on experience with self-testing)
Chai 2010, USA [38]	Chlamydia,gonorrhoea,trichomonas	Urine, penile swab	Illustrated written	Home (Internet request for postal delivery)	M ≥ 14 yrs(median 24.5 yrs)	501 (476)	30%	SAQ (Postal)
Dodge 2010, USA [17] Rosenberger 2011, USA [18]	Chlamydia, gonorrhoea	Rectal swab	NR	A STD clinic, 7 community locations (HIV/AIDs service organization, all-male bathhouse, 2 ‘gay-oriented’ bars, 3 black/Latino-community based organisations)	M 18–57MSM only	68 (75)	NR	IAQ before (n = 75) and after SS (n = 68)
Graseck 2010, USA [49]	Chlamydia,gonorrhoea	Vaginal swab	Home group: detailed written instruction with photographClinic group: Illustrated written instructions (self-sampling) or normal practice of clinic	Home or clinic(number not stated) Participants given the option to choose either home or clinic, the latter include one of four family planning clinics or clinic of the participant’s choice	F 14–45 yrsParticipants of Contraceptive CHOICE project	228 (403)228 samples at 12months follow-up; all 403 expressed opinion had self-sampled at baseline enrolment	57% at follow-up	TIAQ 12 months after enrolment (only asked for preference of follow-up screening)
Graseck 2010, USA [39]	Chlamydia, gonorrhoea	Vaginal swab	Home group: detailed written instruction with photograph Clinic group: Illustrated written instructions (self-sampling) or normal practice of clinic	Home or clinic (number not stated) Randomised into home or clinic groups, the latter can choose one of four family planning clinics or clinic of the participant’s choice	F 14–45 yrs Participants of Contraceptive CHOICE project	243 (122)Home 151Clinic 92Of the 122 expressed opinion, 76 used self-swab	44%	SAQ 56 days after 12 months follow-up testing was offered
Kimmitt 2010, UK [40]	Chlamydia, gonorrhoea	Tampons	Written Instructions	GU outreach clinic or sex workers’ place of work	F Sex workersAge NR	63 (63)	97%	SAQ following SS (n = 63), some also undergo CS (n = 61) afterwards
Greenland 2011, Netherlands [5]	Chlamydia	Vaginal swab, Urine	Information leaflet	At home	M and F 16–29 yrs, sexually active	3508 (3499)	63%	Online questionnaire administered through the internet based programme post SS
Huppert 2011, USA [19]	Trichomonas	Vaginal swab	Illustrated written	Urban pediatric hospital’s Teen Health Center or ED	F14–22 yrs and reporting sexual intercourse in preceding 6 month	274 (247)	90%	SAQ (containing 2 item) pre and post SS, and a third survey post discussion of results
Freeman 2011, USA [46]	Chlamydia, gonorrhea	Pharyngeal swab	Provided instruction (not clear verbal or written), an accompanying diagram and a mirror	STD clinic	MSM undergoing testing for CT and NGAge NR	480 (471)	55.2%	SAQ immediately after SS; (Questionnaire based on Wayal et al 2009
Huppert 2012, USA [20]	Bacterial vaginosis	Vaginal swab	Illustrated written	Urban Children Hospital	F 14–22 yrs reporting sexual intercourse in preceding 6 month	131 (131)	53%	SAQ (containing 2 item) pre and post SS, and a third survey post discussion of results
Reagan 2012, USA [41]	Chlamydia, gonorrhoea	Urine	Step by step written instruction with photograph	Home (n = 100) or clinic (n = 100) Randomised into SS at Home or SS at Clinic	M 18–45 yrs	200 (129)	60%	TIAQ 10–12 weeks post enrolment
Kwan 2012, Australia [53]	Chlamydia	Urine for male, vaginal swab for female	Written instruction downloaded online	At home	M and F 16–63 yrs	377 (55)	56%	Online satisfaction survey after SS
Kohli 2013, Kenya [59]	Chlamydia	Vaginal swab	NR	Two hospital sites	F18–45 yrsSexually active and attending outpatient clinics and A&E departments	300 (300) Self-collection(n = 105)Collection by Health care professionals (n = 195)	NR	NR
Roth 2013, USA [43]	Chlamydia, gonorrhoea,trichomonas	Vaginal swab (100%), rectal swab (50%), oropharyngeal swab (4.5%)	Verbal instruction	NR	M and F 19–65 yrs Recently engaged in transactional sex	44 participants; 24 F and 20 M (44)	100%	FID immediately after SS(mainly about their experience with self obtained sample)
Gudka 2013, Australia [44]	Chlamydia	Vaginal swab	Written instructions leaflet	Recruited from 20 community pharmacies At home or pathology laboratory	F aged >18 yearsAsymptomatic requestingemergency contraception,	166 (91)	28%	Computer assisted telephone interview 3 to 6 weeks post SS
Gaydos 2013, USA [45]	Trichomonas, chlamydia, gonorrhea	Genital and/or rectal swab	Written leaflet and online instruction	At home	F ≥15 yearsCase control study: Cases (having ever used SS kit) Control (never using the kit	Cases = 304Control = 608	NR	SAQ (Postal)
Fielder 2013, USA [52]	Trichomonas, chlamydia, gonorrhea	Vaginal swab	Provided verbal and written instructions leaflet	University Health Service Centres	Female, ean age (18.1, SD = 0.3)First year undergraduate student	310 (413)	64%	SAQ immediately after SS

M: Male; F: Female, MSM: Men having sex with men, GUM: Genitourinary Medicine Clinic, GP: General Practitioner, ED: Emergency Department, OPD; Outpatient Department, NR = Not Reported; SAQ = Self-administered Questionnaire; IAQ = Interviewer-administered Questionnaire; FID = Face-to-Face Interview Discussion; TIAQ = Telephone Interviewer-administered Questionnaire; SS = Self-Sample; CS = Clinician-Sample

## Results

A total of 558 references were initially identified from the computerized search but 410 were excluded after screening the titles. Abstracts of the remaining 148 articles were retrieved for further scrutiny, and after reviewing the abstracts, 45 studies described in 47 articles were found to be eligible for inclusion (Table 1).

Overall, the majority of studies were conducted in Western countries; 24 studies were conducted in North America (23 USA, 1 Canada), 13 in Europe (9 UK, 4 Netherlands), 2 in Asia (1 Thailand, 1 India), 2 Oceania (Australia), 3 in Africa (2 in South Africa and 1 in Kenya), and 1 in South America (Brazil). The location of self-sampling varied between studies; 23 studies were conducted in clinical settings, 10 in home/community settings, 3 across clinical and non-clinical settings, 3 in government establishments (military forts, federal prisons or juvenile correctional facilities), 4 randomised into either home or clinic settings, and the locations for 2 were not reported. The age range of the populations in the included studies was 12–71 years old. In most studies (n = 31), participants were given written instructions with or without illustrations or verbal explanations for their corresponding self-sampling procedures, 6 studies provided only verbal instructions, and the means of explanation was either not published or unclear in the remaining eight studies.

There were large variations in response rate (defined as number of samples/ number of eligible participants) of the studies ranging from 18% to 100%. In general, studies utilizing postal means of recruiting participants tended to have lower response rates compared to studies recruiting in sexually transmitted disease (STD) clinics or other clinical environments. Information on patient’s acceptability was commonly gathered via self-administered questionnaires (SAQ) (n = 27) or interviewer-administered questionnaires (IAQ) (n = 7) or telephone interviewer-administered questionnaires (TIAQ, n = 4) or face-to-face interview discussions (FID) (n = 3) using only open-ended questions on a small number of selected participants. Two studies used a mixture of methods (SAQ & IAQ or SAQ & FID) and two studies did not report the means of collecting information on acceptability. In most studies, these questionnaires or interviews were administered at the end of the study immediately after the participants performed self-sampling with or without undergoing clinician sampling, a few sought participants’ opinion both before and after self-sampling [16,17,18,19,20].

### Outcome measures

There were considerable variations in how acceptability and experience were measured and reported. Patients were commonly asked to comment on the ease of use, confidence, satisfaction or other factors related to the method of self-sampling. The results are described below.

#### Acceptability and experience

Thirty-six studies recorded participants’ experience of or opinion on self-sampling [3,5,16,17,19,20,21,22,23,24,25,26,27,28,29,30,31,32,33,34,35,36,37,38,39,40,41,42,43,44,45,46,47,48,49,50] (S1 Table). Pooled results from these studies shows that self-sampling is a highly acceptable method with 85% of patients reporting the method to be well-received and acceptable (Table 2). Seventeen studies described participants’ desire to self-sample rather than clinician sampling [16,17,24,25,27,29,30,31,32,35,36,38,40,43,45,46,47]. Seven of these compared participants’ preferences for self- versus clinician- administered sampling after undergoing both procedures [25,29,32,35,36,45,46]. Five studies showed a strong preference for self-sampling [25,29,36,45,46]. The participants’ preferences in the other two studies were less conclusive showing only approximately half of the participants desiring self-sampling [32,35]. A strong preference for clinician-sampling was justified by participants’ perception of physicians as experts in sample taking [35]. An additional ten studies compared participants’ preferences after performing self-sampling against hypothetical physician-sampling and showed a strong preference for the former option [16,17,24,27,30,31,38,40,43,47]. Some participants with no preferences were in favour for the most accurate test whichever it may be [27]. One study compared self-sampling using oral-throat rinse with a hypothetical vignette of a physician taken pharyngeal swab and found participants had mixed views with approximately half choosing each option [34]. In Gaydos et al.[30] participants’ who did not submit a self-sample were also surveyed about their preference. They showed a stronger preference for physician examination compared to those who submitted a self-sample (25% vs. 13%).

**Table 2:** Patients’ experience of collecting self-sample*.

Patients’ Experience	Study
**Acceptability and experience (%)**	
Mean 85 SD (15) Median 90 Range (29–100)	Bloomfield 2003 [26]; Brown 2010 [37]; Chai 2010 [38]; Chernesky 2005 [29]; Dodge 2010 [17]; Freeman 2011 [46]; Gaydos 2006 [30]; Gaydos 2013[45]; Gotz 2005 [50]; Graseck 2010 [49]; Graseck 2010 [39]; Greenland 2011 [5]; Hoebe 2006 [31]; Holland-Hall [24]; Hsieh 2003 [48]; Huppert 2011 [19]; Jones 2007 [33]; Kimmitt 2010[40]; Lippman, 2007 [3]; Macmillan 2000 [22]; Markos 1994 [21]; Newman 2003 [27]; Papp 2007 [34]; Pimenta 2003 [28]; Reagan 2012 [41]; Richardson 2003 [16]; Roth 2013 [43]; Tanksale 2003 [42]; Van der Helm 2009 [35]; Van-de-Wijgert 2006[32]; Wayal 2009 [36]; Wiesenfeld 2001[47]
**Ease of Sampling (%)**	
Mean 88 SD (11) Median 93 Range (60–100)	Bloomfield 2003 [26]; Chai 2010 [38]; Chernesky 2005 [29]; Fielder 2013 [52]; Freeman 2011 [46]; Gaydos 2006 [30]; Gotz 2005 [50]; Graseck 2010 [39]; Greenland 2011[5]; Hoebe 2006 [31]; Holland-Hall 2002 [24]; Hsieh 2003 [48]; Jones 2007 [33]; Kimmitt 2010 [40]; Kwan 2012 [53]; Lippman, 2007 [3]; Macmillan 2000 [22]; Mahilum Tapay 2007 [51]; Newman 2003 [27]; Papp 2007 [34]; Pimenta 2003 [28]; Reagan 2012 [41]; Roth 2013 [43]; Tanksale 2003 [42]; Van der Helm 2009 [35]; Van-de-Wijgert 2006 [32]; Wiesenfeld 2001 [47]
**Pain and Discomfort (%)**	
**Pain** Mean 13 SD (15) Median 4 Range (0–42)	Hsieh 2003 [48]; Jones 2007 [33]; Roth 2013 [43]; Tanksale 2003 [42]; Van-de-Wijgert 2006 [32]
**Discomfort** Mean 13 SD (9) Median 10 Range (0–32)	Berwald 2009 [55]; Bloomfield 2002 [54]; Brown 2010 [37]; Fielder 2013 [52]; Hsieh 2003 [48]; Lippman, 2007 [3]; Macmillan 2000 [22]; Markos 1994 [21]; Newman 2003 [27]; Roth 2013 [43]; Van der Helm 2009 [35]; Wayal 2009 [36]
**Confidence in sampling and trust in Results (%)**	
Mean 84 SD (9) Median 84 Range (64–95)	Dodge 2010 [17]; Gaydos 2013 [45]; Jones 2007 [33]; Lippman 2007 [3]; Markos 1999 [21]; Newman 2003 [27]; Stephenson 2000 [23]; Tanksale 2003 [42]
**Concerns and Worries (%)~**	
**Privacy** Mean 30 SD (19) Median 26 Range (4–55)	Bloomfield 2002 [54]; Bloomfield 2003 [26]; Dodge 2010 [17]; Gaydos 2013 [45]; Graseck 2010 [49]; Gudka 2013 [44]; Stephenson 2000 [23]
**Safety** Mean 17 SD (18) Median 18 Range (2.0–43)	Bloomfield 2002 [54]; Bloomfield 2003 [26]; Chai 2010 [38]; Gaydos 2013 [45]; Gaydos 2006 [30]
**Sampling preference (%)**	
**Vaginal Swab** Mean 60 SD (21) Median 55 Range (31–94)	Chernesky 2005 [29]; Fielder 2013 [52]; Gaydos 2006 [45]; Greenland 2011 [5]; Holland-Hall 2002 [24]; Hsieh 2003 [48]; Jones 2007 [33]; Kohli 2013 [59]; Macmillan 2000 [22]; Newman 2003 [27]; Tebb 2004 [58]
**Urine** Mean 49 SD (26) Median 45 Range (9.0–95)	Chernesky 2005 [29]; Fielder 2013 [52]; Gaydos 2006 [30]; Greenland 2011 [5]; Holland-Hall 2002 [24]; Hsieh 2003 [48]; Jones 2007 [33]; Kohli 2013 [59]; Macmillan 2000 [22]; Mahilum Tapay 2007 [51]; Newman 2003 [27]; Tebb2004 [58]
**Clinic or home? (%)**	
**Clinic** Mean 44 SD (23) Median 42 Range (0.0–78)	Graseck 2010 [39]; Graseck 2010 [49]; Hsieh 2003 [48]; Jones 2007 [33]; Kimmitt 2010 [40]; Lippman, 2007 [3]; Reagan 2012 [41]; Tebb 2004 [58]; Van-de-Wijgert 2006 [32]
**Home** Mean 65 SD (20) Median 60 Range (24–100)	Graseck 2010 [39]; Graseck 2010 [49]; Hsieh 2003 [48]; Jones 2007 [33]; Kimmitt 2010 [40]; Lippman, 2007 [3]; Reagan 2012 [41]; Tebb 2004 [58]; Van-de-Wijgert 2006 [32]
**Willingness to Use or Recommend of Self-Sampling (%)**	
Mean 86 SD (14) Median 92 Range (50–100)	Chandeying 2004 [60]; Chernesky 2005 [29]; Dodge 2010 [17]; Fielder 2013 [52]; Gaydos 2006 [30]; Graseck 2010 [39]; Greenland 2011 [5]; Hoebe 2006 [31]; Holland-Hall 2002 [24]; Jones 2007 [33]; Kimmitt 2010 [40]; Kwan 2012 [53]; Macmillan 2000 [22]; Papp 2007 [34]; Pimenta 2003 [28]; Reagan 2012 [41]; Roth 2013 [43]; Van der Helm 2009 [35]; Van-de-Wijgert 2006 [32]; Wayal 2009 [36]; Wiesenfeld 2001 [47]
**Reasons for Declining or Refusing to Self-Sample (%)**	
**Lack of Time** Mean 25 SD (17) Median 21 Range (7–62)	Fielder 2013 [52]; Graseck 2010 [39]; Greenland 2011 [5]; Gudka 2013[44]; Lippman 2007 [3]; Macmillan 2000 [22]; Richardson 2003 [16]; Serlin 2003 [25]; Tebb 2004 [58]
**Discomfort/Dislike** Mean 25 SD (8) Median 17 Range (2.0–60)	Fielder 2013 [52]; Macmillan 2000 [22]; Macmillan 2000 [22]; Newman 2003 [27]; Serlin 2002 [25]; Van der Helm 2009 [35]; Richardson 2003 [16]
**Not being at risk** Mean 36 SD (8) Median 40 Range (24–43)	Gudka 2013 [44]; Greenland 2011 [5]; Fielder 2013 [52]
**Recently tested** Mean 36 SD (33) Median 20 Range (12–93)	Graseck 2010 [39]; Graseck 2010 [49]; Graseck 2010 [5]; Gudka 2013 [44]
**Menstruation** Mean 11 SD (13) Median 2 Range (2.0–29)	Hoebe 2006 [31]; Kimmitt 2010 [40]; Macmillan 2000 [22]

*% not reported in some studies (see S1 Table for details)

#### Ease of sampling

Twenty-eight studies recorded participants’ views on the ease of use of self-sampling methods[3,5,22,24,26,27,28,29,30,31,32,33,34,35,36,38,39,40,41,42,43,46,47,48,50,51,52,53] (S1 Table). On average, 88% of patients reported that self-sampling was ‘very easy’, ‘easy’ or ‘not difficult’ to perform (Table 2). Two studies [27,51], investigated the ease of collection among other different self-sampling methods. In these studies, the majority of participants found no difference in ease of collection between self-administered vaginal swabs and urine sampling. Interestingly, in a study involving female military recruits in the US, Hsieh et al [48] reported that white army recruits generally found self-administered vaginal swabs easier compared to the black recruits.

#### Pain and discomfort

The experience of pain or discomfort was reported in seventeen studies [3,21,22,25,27,32,33,35,36,37,42,43,46,48,52,54,55] (S1 Table). A small proportion of participants (13%) complained of these negative experiences (Table 2). Fielder et al [52] reported that on average, women felt comfortable, relaxed and in control while obtaining the vaginal swab and strongly disagreed that obtaining the sample was painful. When comparing various types of sampling methods, Hsieh et al. [48] reported that women found self-administered vaginal swabs significantly more painful than urine collection (2% vs.0.1%; p<0.05). A randomised controlled trial (RCT) demonstrated that, despite using the same sampling method, participants felt more pain self-sampling at home (17%) when compared to self-sampling in a clinic (12%) [33]. When comparing the experiences of self-sampling (urine for men, vaginal swab for women) and clinician-sampling (urethral swab for men and speculum examination and endocervical swabs in women) in the same setting, one study indicated self-sampling was less painful or less uncomfortable than clinician-sampling [37]. A further study conducted in clinical setting reported that the proportion of participants experiencing pain during clinician examination is much greater (15%) than those self-sampling (3%) with tampons or vaginal swabs [32]. In contrast, another study conducted in a clinical setting comparing the same sampling methods reported stronger preferences towards clinician collected samples (60%, n = 45) as participants felt that self-administered swabs were uncomfortable and painful [42].

#### Confidence in sampling and trust in test results

Thirteen studies assessed participants’ level of confidence in obtaining self-samples or interpreting the results [3,17,19,20,21,23,25,27,33,36,42,45,52] (S1 Table). Studies shows that majority of the participants (84%) expressed confidence in doing the tests properly or believed they had taken the sample/ interpreted the results correctly. Serlin et al.[25] found that despite self-sampling being the preferred method, participants trusted the results of a physician pelvic examination the most, followed by urine sampling, and self- vaginal swabbing the least. Studies by Huppert et al. [19,20] reported lower trust in self-testing compared to clinician testing at baseline, with the trust increasing after testing experience. After the discussion of the results, trust of self and clinician testing did not differ significantly [20]. Gaydos et al [45] also collected opinions from those who performed the self-administered swab, with controls, who never performed self-sampling. Interestingly, it was found that those who performed the test were more likely to think that the sampling procedure was accurate compared to the controls (83% vs 63%).

#### Concerns and worries

Ten studies addressed, to a certain degree, participants’ concerns and worries regarding self-sampling [17,23,26,28,30,38,39,44,45,54] (S1 Table). Privacy and safety regarding self-collection of sample was the commonest issue of concern with 30% of patients being concerned about privacy and 17% regarding safety (Table 2). In a study from the USA, consisting of mainly male participants, Bloomfield et al. [54] reported that 56% very worried about confidentiality, 54% about privacy, and 36% safety of screening using urine test kits sent through the mail. The same group conducted further research [26] targeting both genders which concluded that men (except men having sex with men (MSM)) are most likely to voice concerns about self-sampling, followed by women, and MSM were the least likely to raise any concerns. However, neither paper defined whether privacy meant ‘anonymity/confidentiality of samples’ or ‘privacy during self-sampling’. Two studies recorded negative comments concerning the delivery of the self-sampling method [17,28]. In Dodge et al. [17], MSM reported that rectal swab self-sampling in community based venues lacked privacy, accuracy and sterility. Some participants (proportion not reported) in the study by Pimenta et al. [28] expressed dissatisfaction about the handing out of containers and collecting specimens at the front desk by the receptionist, instead of managing the specimen handling more discreetly.

#### Demographic characteristics

The characteristics of participants performing or willing to perform self-sampling were reported in fewer studies. Only three studies looked at the acceptability of self-sampling by gender and found that females were more likely to accept, or be willing to self-sample in the future [5,39,50]. Eight studies reported that older participants were more willing to self-sample [3,19,27,28,41,48,49,56], three reported no difference by age [29,37,57] and two reported older participants being less likely to choose self-sampling compared to younger participants [5,32]. In three studies, level of educational attainment did not influence acceptability of self-sampling [29,32,57]. One study, however, reported that low educational level was significantly associated with increased willingness to test [5]. In some studies that looked at differences between Caucasians and other ethnic minorities, it was noted that the former group was more likely to accept, be comfortable with, and prefer self-sampling [39,48,54,56].

#### What type of self-sampling?

Participants’ preferences towards self-sampling using urine or self-administered vaginal or vulval swabs was assessed in 15 studies [5,22,24,25,27,28,29,30,33,34,48,51,52,58,59] (S1 Table). A pooled result from these studies shows that the preference for vaginal swab was slightly higher than urine (60% vs 49%). In a study by Pimenta et al. [28], the majority of women commented they would not have participated in the study had there been a need to perform a vaginal swab. In a study involving military recruits, Hsieh et al. [48] further explored whether women’s preferences would change should the location of self-sampling vary (at home, in clinic, or in the field). It was found that the majority of women’s sampling preference remained unchanged between locations.

#### Clinic or home?

Participants’ opinions on the preferred location for self-sampling was assessed in 11 studies [3,5,32,33,39,40,41,46,48,49,58] (S1 Table). Findings from these studies suggest that participants prefer to self-sample at home (65%) than in clinic (44%) (Table 2). In Regan et al. [41], men assigned to a home sampling kit were 60% more likely to complete screening compared to men assigned to clinic screening. Common reasons given for such preferences included ‘less embarrassment’, ‘convenience’, and ‘long waiting time’ in clinics [40]. In one study, only a small proportion (12%) of the women found home testing an unpleasant concept [5]. In another study based in South Africa [32], although the majority believed women should be given the option to obtain the sample at home and bring to the clinic, most women expressed a desire to self-sample in the clinic. Three recent RCTs yielded mixed conclusions [3,33,39]. Two of these demonstrated women’s preferences were highly associated with their randomisation groups [3,33]. The majority of participants preferred to self-sample in the setting where they had the testing experience (i.e. home group preferred home self-sampling while the clinic group preferred clinic self-sampling). In the third RCT study [39], it was reported that while the majority in the home group expressed a preference for home self-sampling, only half of those in the clinic group chose clinic self-sampling.

#### Willingness to use or recommend of self-sampling?

Patients’ willingness to use self-sampling again in the future was assessed in 21 studies [5,17,22,24,28,29,30,31,32,33,34,35,36,39,40,41,43,47,52,53,60]. These studies found that majority of the patients (86%) were willing to use the self-sampling methods in the future. Willingness to use self-sampling seemed to apply to participants who were, prior to the study, naïve to using self-swabs [38]. Using self-sampling methods, most participants would test more often [5,29,47]. One study conducted in the USA reported that participants were willing to test between their regular pelvic exams [24]. An African study by van de Wijgert et al. [32] found that participants were more willing to self-sample at home than in the clinic. Two additional clinic-based studies reported patients, after being offered and undergoing self-sampling on that occasion, would be happy to undergo self-sampling in the future [35,36,57]. In an American study by Gaydos et al. [30] 91% of the ‘questionnaire only’ submitters reported willingness to use further self-sampling method compared to 86% test kit users. Five additional studies showed participants would recommend or encourage family and friends to self-sample [17,22,28,41,43]. Current STI infection, female gender, younger age group, low education and having two or more sexual partners in the last 6 months were significantly associated with increased willingness to self-test in the future [5].

### Reasons for Declining or Refusing to Self-Sample

Sixteen studies recorded the reasons given by potential participants who refused to self-sample [3,5,16,17,22,25,27,31,35,36,39,40,44,49,52,58]. The most common reason was a lack of time [3,5,16,22,25,44,49,52,58] or dislike/discomfort with the concept of vaginal swab/self-sampling [16,22,25,27,35,36,52], followed by the perception of not being at risk, recently being tested elsewhere and current menstruation. Other reasons included general lack of interest fear of incorrect self-sampling technique, virginity, inability to understand the instructions/information, lack of confidentiality, sanitary issues, the perception that they were not at risk for an STI or not wanting to know if they had an STI.

### Critical Appraisal of Study Quality

The study quality was assessed using criteria included in the STROBE statement [15] (S1 Table). All studies (45) had an abstract. Four studies (9%) did not clearly describe their recruitment or participant selection methods [25,27,49,50], four (9%) did not socio-demographic details of the study population [21,34,40,52] and eight (18%) did not publish or clearly state their means of providing self-sampling instructions (i.e. whether it was written, verbal, or video) [17,25,27,28,37,56,58,59]. Two studies did not report their means of ascertaining participants’ opinion [3,59] and thirteen did not provide the source of funding [21,23,26,31,34,35,40,43,46,53,54,55,59].

## Discussion

To our knowledge, this is the first systematic review summarising patients’ experiences of obtaining self-samples to diagnose curable STIs. Reviewing 45 studies, it was found that patients found self-sampling highly acceptable. The majority of participants who took part found it very easy to collect the self-sample, which ranged from urine samples to oral gargle and were willing to self-sample again in the future, and also to recommend self-sampling to family and friends. Most did not have any unpleasant experiences performing the self-sampling procedures, and preferred self-sampling to physician sampling. These results are consistent with previous review focussing on HPV self-sampling [11,12] and HIV self-testing [13].

Consistent with a previous review by Shih et al [61], we found that most patients preferred to self-sample at home rather than in the clinic. Interestingly, it was found that people are more compliant if the mode of testing reflects their preferences. In a recent study by Graseck et al. [49], patients choosing to self-sample at home were twice as likely to actually complete the test compared to those choosing the clinic option (where both self-sampling and non-self-sampling means were offered). Therefore, one may regard this higher ‘compliance rate’ as a secondary advantage of home-based self-screening over and above women’s choice. In recent years self-sampling has emerged as an important tool in screening as well as being used as a routine means of diagnostic sampling in a clinical setting. Professionals hold the belief that not only does this reduce the workload of staff, but it also provides patients with greater autonomy, privacy, and confidentiality [62]. However, this perspective is challenged by some patients. Women in one of our included studies stated that should self-sampling be adopted instead of their annual physician appointment, they would rather continue with pelvic examinations. This view is further confirmed by a recent large survey (n = 2,887) across three American clinic sites by Howard et al [63]. The authors reported, as an alternative to self-sampling, the majority would prefer to wait to see a doctor, even if there is a hypothetical long wait and a substantial number would prefer to come back the next working day should the clinic turn patients away. A recent UK study explored patients’ desire for clinician genital examination. Basta et al [64] found that most asymptomatic patients (98% female, 91% male) presenting to the GUM clinic were in favour of routine genital examination. The participants in this study had not knowingly been in contact with any STI and there were no statistical difference in preferences between patients surveyed before or after undergoing clinician examination. Moreover, this preference was consistent across all age groups, even the youngest age group (16–19 years) who are usually presumed to preclude intimate examinations because of embarrassment or discomfort. The conclusions from these two studies cannot be generalized as neither looked into the reason for such preferences or sought opinion beyond clinic attendance. Nevertheless they highlight the importance of educating patients about the limited evidence of the benefits of genital examination whilst stressing the effectiveness of self-sampling, in order to enable patients to make informed choices.

This systematic review identifies a lack of standardization for self-sampling methods, and the use of different analytic methods for STI detection and sampling procedures make it difficult to make any suggestion regarding what should be considered as the best self-sampling practice. The settings of the studies ranged from specialist centres to non-healthcare settings, with the majority of studies conducted in non-specialist healthcare settings. Some (n = 7) studies were conducted in sexual health clinics and these participants may be a subgroup of regular clinic attendants who have been empowered, over years of experience, to take more initiative in self-care and held different views compared to the normal population. However, as only a minority of the studies were conducted in such specialist settings, we believe that findings of this review are generalizable to wider settings. The majority of studies used either self-completed or interviewer-administered questionnaires to seek participant’s opinions regarding self-sampling, but only a few (n = 5/45) provided the exact wording of their survey instrument. Hence, it is not clear whether these questionnaires had elicited patients’ opinions as intended. Unfortunately, there is no reliable standard to measure outcome of ‘opinion questionnaires’ such as patient acceptability. Rigorous attempts should be made to ensure the validity of these questionnaires. Furthermore, in the case of interviewer-administered questionnaires, patients’ ideas may be subject to the researchers’ interpretation. The use of self-administered questionnaires with an open-ended option may be a preferable option. Some studies [30] [5] also gathered the opinions of participants who refused to self-sample which has highlighted some important potential negativities of self-sampling. This bias is further compounded by the fact that some studies have surveyed/interviewed fewer participants than sampled [28]. Patients’ views in these studies were only partially sought or acceptability only investigated as part of a larger study. Patients who did not accept the self-sampling offer are likely to have different opinions which were mainly undocumented.

One might anticipate that socio-demographic and cultural factors would influence acceptance of and preferences for self-sampling. It is therefore, disappointing that not all studies collected comprehensive socio-demographic details. A certain degree of variability existed between studies conducted in different cultural and racial groups. A high response rate, where reported, was generally found in ‘Non-Western’ (i.e. Non-European/Non-North American) studies. This figure is particularly high in the studies conducted in Asia [60] (94%) or South America [3] (84%) compared to the African study (45%) [33]. Potential differences in opinion between ethnic groups have also been investigated by focus groups, where actual self-sampling did not take place. For example, Forrest et al. [65] reported the attitudes of women of Indian, Pakistani, African-Caribbean and of white British origin residing in the UK and found that the majority of women had strong intentions to use self-test for HPV. Another focus group study by Howard et al [66] examined the barriers to self-sampling in six ethnic groups (Afghani, Somali, Arabic, Chinese, Hispanic, and Canadian) residing in Canada. In these studies, despite participants generally appreciating the benefits of self-sampling, all expressed a certain degree of reservation.

The strength of this systematic review lies in the assessment of the reporting of the studies using STROBE checklist. However, this review is subject to a number of limitations. We did not include the studies published in language other than English which may have introduced bias in the findings of this review. Nevertheless, this review did include studies from countries such as India, Thailand, and Brazil. In addition, cost-effectiveness of self-sampling was not addressed in this review. However, we noted that two studies looked at the cost per specimen received [26,54], one study calculated a net profit of self-testing over clinic-testing [38], and two studies looked at the cost that participants were willing to pay for self-sampling [3,33,67]. The anticipated cost for the health services is important for STI self- sampling and future studies need to conduct economic evaluations of self-sampling to develop an effective policy for the management of STIs.

In conclusion, despite the heterogeneity among studies, particularly of screening methods, populations studied, settings of self-sampling and methods of ascertaining acceptability, this systematic review has demonstrated that self-sampling is a well-accepted and preferred approach to test for STIs. In the current context of low uptake of STI testing, using home/community-based self-sampling method could be a promising approach to increase participation among hard-to-reach populations, and to reduce sampling related barriers such as stigma, confidentiality and inconvenience. Although, acceptance or preference for self-sampling does not necessarily reflect actual testing behaviour, this review suggests that self-sampling may be a feasible option for those who are hesitant to undergo clinician sampling.

## Supporting Information

S1 Table Patients’ acceptability and experience of collecting self-sample. (DOCX)

S2 Table Study quality assessment using STROBE guidelines. (DOCX)
