# Supplementary material for: Obtaining Self-Samples to Diagnose Curable Sexually Transmitted Infections: A Systematic Review of Patients’ Experiences
Source: PLoS One. 2015 Apr 24;10(4):e0124310. doi: 10.1371/journal.pone.0124310 (PMC4409059; doi:10.1371/journal.pone.0124310)
Supplement: S1 Table — (DOCX) [file pone.0124310.s001.docx]

| **Study** | **Acceptability and experience** |
| --- | --- |
| Bloomfield 2003 [[26](#_ENREF_26)] | 83% found SS acceptable |
| Brown 2010 [[37](#_ENREF_37)] | 90% of men reported good experience and 70% reported comfortable, 80% of women reported the process as comfortable |
| Chai 2010 [[38](#_ENREF_38)] | 77% preferred SS |
| Chernesky 2005 [[29](#_ENREF_29)] | 76% preferred SS |
| Dodge 2010 [[17](#_ENREF_17)] | 72% preferred SS |
| Freeman 2011 [[46](#_ENREF_46)] | 92% willing to SS at home |
| Gaydos 2006 [[30](#_ENREF_30)] | 89.5% preferred SS |
| Gaydos 2013 [[45](#_ENREF_45)] | 93% preferred SS |
| Gotz 2005 [[50](#_ENREF_50)] | 97% feel fine to do at home |
| Graseck 2010 [[39](#_ENREF_39)] | 81 % very or somewhat satisfied |
| Graseck 2010 [[49](#_ENREF_49)] | 75.7% choose screen for STI at home |
| Greenland 2011 [[5](#_ENREF_5)] | 96.9% found home sampling advantageous |
| Gudka 2013 [[44](#_ENREF_44)] | Highly convenient (% not reported) |
| Hoebe 2006 [[31](#_ENREF_31)] | 100% felt confident performing SS |
| Holland-Hall 2002 [[24](#_ENREF_24)] | 95.5% liked self-testing |
| Hsieh 2003 [[48](#_ENREF_48)] | Acceptability of urine at home 90.9%, in the field 79.4%; acceptability of vaginal swab at home: 82.9%, in the field: 68.8% |
| Huppert 2011 [[19](#_ENREF_19)] | 93% willing to test at home |
| Huppert 2012 [[20](#_ENREF_20)] | % not reported |
| Jones 2007 [[33](#_ENREF_33)] | 96% comfortable collecting their own swab |
| Kimmitt 2010 [[40](#_ENREF_40)] | Tampon sampling preferred by 95% |
| Lippman, 2007l [[3](#_ENREF_3)] | 96% comfortable with SS |
| Macmillan 2000 [[22](#_ENREF_22)] | Vulval swab acceptable to 93.2% and urine to 98.1% |
| Markos 1994 [[21](#_ENREF_21)] | 88% reported SS to be comfortable |
| Newman 2003 [[27](#_ENREF_27)] | 60% preferred SS, 23% preferred a pelvic examination |
| Papp 2007 [[34](#_ENREF_34)] | 93% acceptable with Mouth wash and 95 % with Water |
| Pimenta 2003 [[28](#_ENREF_28)] | 100% found urine sample acceptable |
| Reagan 2012 [[41](#_ENREF_41)] | 80% very satisfied with screening |
| Richardson 2003 [[16](#_ENREF_16)] | 54.2% mildly or strongly preferred SS |
| Roth 2013[[43](#_ENREF_43)] | 88% of women and 90% of men preferred SS |
| Serlin 2002 [[25](#_ENREF_25)] | Data not reported in % |
| Stephenson 2000 [[23](#_ENREF_23)] | No negative response regarding acceptability (% not reported) |
| Tanksale 2003 [[42](#_ENREF_42)] | 60% preferred CS, 29% preferred SS and 11% felt both method acceptable |
| Van der Helm 2009 [[35](#_ENREF_35)] | SS comfortable to 91% of MSM and 81% of women |
| Van-de-Wijgert 2006 [[32](#_ENREF_32)] | 63.3% reported good experience of SS |
| Wayal 2009, UK [[36](#_ENREF_36)] | Overall acceptance 84% |
| Wiesenfeld 2001 [[47](#_ENREF_47)] | 83% preferred SS |

| **Study** | **Ease of sampling** |
| --- | --- |
| Bloomfield 2003 [[26](#_ENREF_26)] | 11% reported difficulty using the test kit |
| Chai 2010 [[38](#_ENREF_38)] | 89% reported very easy or easy |
| Chernesky 2005 [[29](#_ENREF_29)] | 96.2% found collecting vaginal swab very easy |
| Fielder 2013 [[52](#_ENREF_52)] | 93% found SS easy or very easy |
| Freeman 2011 [[46](#_ENREF_46)] | 80% reported easy to collect |
| Gaydos 2006 [[30](#_ENREF_30)] | 90.5% found SS very easy or easy |
| Gotz 2005 [[50](#_ENREF_50)] | 65% said urine collection easy process |
| Graseck 2010 [[39](#_ENREF_39)] | 73% reported SS an easy procedure |
| Greenland 2011 [[5](#_ENREF_5)] | 89% found SS easy |
| Hoebe 2006 [[31](#_ENREF_31)] | 90% reported easy to perform |
| Holland-Hall 2002 [[24](#_ENREF_24)] | 94.7% found self-testing easy |
| Hsieh 2003 [[48](#_ENREF_48)] | 60.4% reported urine easier method, 33.2% choose chose SAS |
| Jones 2007 [[33](#_ENREF_33)] | 85.8% of from home group easy or very easy compared to 96.2 % in clinic group |
| Kimmitt 2010 [[40](#_ENREF_40)] | 100% found it easy |
| Kwan 2012 [[53](#_ENREF_53)] | 97% reported the process to be easy |
| Lippman, 2007 [[3](#_ENREF_3)] | 96% found self-collection easy |
| Macmillan 2000 [[22](#_ENREF_22)] | 94.2% reported SS as an easy procedure |
| Mahilum Tapay 2007 [[51](#_ENREF_51)] | 95.9% comfortable collecting swab |
| Newman 2003 [[27](#_ENREF_27)] | 53% found no difference, 23% reported urine collection easier, and 21% reported swabbing easier |
| Papp 2007 [[34](#_ENREF_34)] | 93% found easy to SS |
| Pimenta 2003 [[28](#_ENREF_28)] | 100% found quick and easy |
| Reagan 2012 [[41](#_ENREF_41)] | 80% reported as a convenient method |
| Roth 2013 [[43](#_ENREF_43)] | 93% said urine and vaginal swab collection easy process |
| Tanksale 2003 [[42](#_ENREF_42)] | 65% preferring SS reported easy to collect |
| Van der Helm 2009 [[35](#_ENREF_35)] | SS easy to 94% of MSM and 95% of women |
| Van-de-Wijgert 2006 [[32](#_ENREF_32)] | 92.6% reported easy or very easy procedure |
| Wayal 2009, UK [[36](#_ENREF_36)] | % not reported |
| Wiesenfeld 2001 [[47](#_ENREF_47)] | 99% reported SS as easy |
| **Pain and discomfort** | |
| Berwald 2009 [[55](#_ENREF_55)] | 100% comfortable with SS |
| Bloomfield 2002 [[54](#_ENREF_54)] | 4% reported sampling difficulty |
| Brown 2010 [[37](#_ENREF_37)] | Clinician-sampling uncomfortable to self-sampling in 33% vs 20% in women and 49% vs 30% in men |
| Fielder 2013 [[52](#_ENREF_52)] | 26% felt uncomfortable with SS |
| Freeman 2011 [[46](#_ENREF_46)] | % not reported |
| Hsieh 2003 [[48](#_ENREF_48)] | Pain on collecting a urine and vaginal swab (0.1%) and (2.2%), discomfort reported by19% and 26.3% respectively. |
| Lippman, 2007 [[3](#_ENREF_3)] | SS uncomfortable to 5% in home group and 4 % in clinic group |
| Jones 2007 [[33](#_ENREF_33)] | 17% in the home group and 12.3% in the clinic group reported pain |
| Macmillan 2000 [[22](#_ENREF_22)] | 4.9% found SS uncomfortable |
| Markos 1994 [[21](#_ENREF_21)] | 1.3% reported high discomfort |
| Newman 2003 [[27](#_ENREF_27)] | 13% of women found swab method uncomfortable |
| Roth 2013 [[43](#_ENREF_43)] | 96% described the process of collecting urine or vaginal swab as painless and 93% as comfortable |
| Serlin 2002 [[25](#_ENREF_25)] | Data not reported in % |
| Tanksale 2003 [[42](#_ENREF_42)] | 42% choosing CS reported self-collection to be painful |
| Van der Helm 2009, [[35](#_ENREF_35)] | SS comfortable to 91% MSM and 87% women |
| Van-de-Wijgert 2006 [[32](#_ENREF_32)] | 3.3% reported pain during SS compared to 15% during CS |
| Wayal 2009, UK [[36](#_ENREF_36)] | Pharyngeal swab collection difficult (24%) and uncomfortable (32.5%); rectal swab collection difficult (19%) and uncomfortable (30.5%) |
| **Confidence in sampling and trust in Results** | |
| Dodge 2010 [[17](#_ENREF_17)] | 18% not confident about accuracy |
| Fielder 2013 [[52](#_ENREF_52)] | % not reported |
| Gaydos 2013 [[45](#_ENREF_45)] | 83% reported self-administered swab to be accurate |
| Huppert 2011 [[19](#_ENREF_19)] | % not reported |
| Huppert 2012 [[20](#_ENREF_20)] | % not reported |
| Jones 2007 [[33](#_ENREF_33)] | 80.5% of from home group and 90.1 % in clinic found easy to read results |
| Lippman, 2007[[3](#_ENREF_3)] | 93% in the home group and 97% in clinic group trusted the results |
| Markos 1999 [[21](#_ENREF_21)] | 78.7% reported high confidence in SS |
| Newman 2003 [[27](#_ENREF_27)] | 5% afraid of doing swab wrong |
| Serlin 2002 [[25](#_ENREF_25)] | % not reported |
| Stephenson 2000 [[23](#_ENREF_23)] | 11% of women uncertain about sampling done correctly |
| Tanksale 2003 [[42](#_ENREF_42)] | 36 % choosing CS swab reported lack of confidence in SS sample |
| Wayal 2009 [[36](#_ENREF_36)] | % not reported |
| **Concerns and Worries** | |
| Bloomfield 2002 [[54](#_ENREF_54)] | 56% very concerned about confidentiality, 54% about privacy and 34% about safety |
| Bloomfield 2003 [[26](#_ENREF_26)] | 52% concerned about confidentiality, 45% privacy and 43% safety |
| Chai 2010 [[38](#_ENREF_38)] | 2% concerned about safety |
| Dodge 2010 [[17](#_ENREF_17)] | 26 % concerned about privacy and 4% about sterility |
| Gaydos 2006 [[30](#_ENREF_30)] | 1.8% concerned about the safety |
| Gaydos 2013 [[45](#_ENREF_45)] | 4.3% concerned about privacy, 97% reported as a safe process |
| Graseck 2010 [[39](#_ENREF_39)] | 95% not concerned about privacy |
| Gudka 2013 [[44](#_ENREF_44)] | 50% reported lack of privacy |
| Pimenta 2003 [[28](#_ENREF_28)] | Concern about confidentiality (% not reported) |
| Stephenson 2000 [[23](#_ENREF_23)] | 25% objected receiving sampling kit at home |
| **Sampling preference** | |
| Chernesky 2005 [[29](#_ENREF_29)] | 76% preferred a vaginal swab to a pelvic examination, and 60% over a urine collection |
| Fielder 2013 [[52](#_ENREF_52)] | 50% preferred vaginal swab, 20% urine swab, 5% pelvic examination |
| Gaydos 2006 [[30](#_ENREF_30)] | 54.3% preferred vaginal swab, 8.8% urine and 12.6% pelvic examination |
| Greenland 2011 [[5](#_ENREF_5)] | Swab kit easy than urine kit (94% vs 85%) |
| Holland-Hall 2002 [[24](#_ENREF_24)] | 39% preferred vaginal swab, 37% preferred urine |
| Hsieh 2003 [[48](#_ENREF_48)] | 90.8% felt comfortable collecting urine and 69.6% collecting vaginal swab |
| Jones 2007 [[33](#_ENREF_33)] | In home group, 39.9% liked using urine and 60.1% liked vaginal swab  In clinic group, 49.3% liked urine and 50.7% liked vaginal swab |
| Kohli 2013 [[59](#_ENREF_59)] | 77.7% preferred vaginal swab to endocervical swab |
| Macmillan 2000 [[22](#_ENREF_22)] | 95.1% preferred urine, 78.6% preferred vulval swab |
| Mahilum Tapay 2007 [[51](#_ENREF_51)] | 40.7% preferred swab, 37.5% preferred urine |
| Newman 2003 [[27](#_ENREF_27)] | 31% preferred vaginal swab, 21% preferred urine |
| Papp 2007 [[34](#_ENREF_34)] | 50.4% preferred mouth wash and 48.7% preferred water |
| Pimenta 2003 [[28](#_ENREF_28)] | Urine preferred to swab (% not reported) |
| Serlin 2002 [[25](#_ENREF_25)] | Urine preferred to vaginal swab and pelvic examination (% not reported) |
| Tebb 2004 [[58](#_ENREF_58)] | 63% Urine by and vaginal swab by 37% |
| **Clinic or home?** | |
| Freeman 2011 [[46](#_ENREF_46)] | Exact % not reported |
| Graseck 2010 [[39](#_ENREF_39)] | Self-sampling convenient to 89% in home group and 69% in clinic group |
| Graseck 2010 [[49](#_ENREF_49)] | Women choosing home base testing more likely to complete a test compared with all clinic based tester (64.6% vs 31.6%) |
| Greenland 2011 [[5](#_ENREF_5)] | 12% found home testing an unpleasant concept |
| Hsieh 2003 [[48](#_ENREF_48)] | Collection of FVU more preferred than swab in the clinic (56.4% vs 32.9%) and at home (52% vs 39.1%) |
| Jones 2007 [[33](#_ENREF_33)] | 58% in home group preferred SS at home and 66% in clinic group preferred doing it in the clinic |
| Kimmitt 2010 [[40](#_ENREF_40)] | 100% favoured testing away from GUM clinic |
| Lippman, 2007 [[3](#_ENREF_3)] | 60% in the home group preferred home collection/testing compared to 42% in the clinic group |
| Reagan 2012 [[41](#_ENREF_41)] | 95% of home group reported the process to be very easy compared with 79% of the Clinic group |
| Tebb 2004 [[58](#_ENREF_58)] | 52% ranked home based urine test as 1^st^ choice compared to 30% who ranked clinic based testing as 1^st^ choice |
| Van-de-Wijgert 2006 [[32](#_ENREF_32)] | 31.1% preferred to SS at clinic, 24% at home and 42.4% by clinician |
| **Willingness to Use or Recommend of Self-Sampling** | |
| Chandeying 2004 [[60](#_ENREF_60)] | 72.6% willing to use tampon and 74.2% willing to use self-administered vaginal swab |
| Chernesky 2005 [[29](#_ENREF_29)] | 94% would like to test in the future |
| Dodge 2010 [[17](#_ENREF_17)] | 90% willing to recommend to their friend |
| Fielder 2013 [[52](#_ENREF_52)] | 73% reported testing more often using SS |
| Gaydos 2006 [[30](#_ENREF_30)] | 86.3% willing to SS in the future |
| Graseck 2010 [[39](#_ENREF_39)] | 83% of home group to test at home again compared 49% of clinic group choosing clinic again |
| Greenland 2011 [[5](#_ENREF_5)] | 66% would test in the future |
| Hoebe 2006 [[31](#_ENREF_31)] | 99% willing to SS again |
| Holland-Hall 2002 [[24](#_ENREF_24)] | 94.7% willing to perform in the future |
| Jones 2007, South [[33](#_ENREF_33)] | 95.1% in home group and 98.5% in clinic group will SS again same setting |
| Kimmitt 2010 [[40](#_ENREF_40)] | 100% would SS again |
| Kwan 2012 [[53](#_ENREF_53)] | 98% would recommend to their friend |
| Macmillan 2000 [[22](#_ENREF_22)] | Willing to recommend vulval swab (78.6%) and urine (89.3%) |
| Papp 2007 [[34](#_ENREF_34)] | 96.8 % willing to SS using mouthwash and and 96.1% using water |
| Pimenta 2003 [[28](#_ENREF_28)] | 100% willing to encourage to friends |
| Reagan 2012 [[41](#_ENREF_41)] | 99% would recommend to their male friend |
| Roth 2013 [[43](#_ENREF_43)] | 87% willing to recommend to their friend |
| Van der Helm 2009 [[35](#_ENREF_35)] | 94% of MSM and 95% of women would use SS again |
| Van-de-Wijgert 2006 [[32](#_ENREF_32)] | 50.1% willing to SS in the future |
| Wayal 2009 [[36](#_ENREF_36)] | 76% willing to use all four method of SS in the future |
| Wiesenfeld 2001 [[47](#_ENREF_47)] | 97% stated testing more often in the future |
| **Reasons for Declining or Refusing to Self-Sample** | |
| Dodge 2010 [[17](#_ENREF_17)] | Data not reported in % |
| Fielder 2013 [[52](#_ENREF_52)] | n=173; scheduling conflict (62%), perception of not being at risk (43), being uncomfortable with swab (26%) |
| Graseck 2010 [[39](#_ENREF_39)] | n=207; forgetting to test (32%), lack of time (16%), not receiving test materials (15%), testing elsewhere (12%), losing or damaging test materials (11%) |
| Graseck 2010 [[49](#_ENREF_49)] | n=12; recently been tested (92.3%) |
| Greenland 2011 [[5](#_ENREF_5)] | n=1263; no perceived risk of infection (40%), no time (9%), recently tested (16%) |
| Gudka 2013 [[44](#_ENREF_44)] | Inconvenience (n=19; 27%), self-assessment of not being at risk (n=17; 24%), testing at the GP instead (n=16; 23%), losing the Kit (n=13; 18%), |
| Hoebe 2006 [[31](#_ENREF_31)] | Current menstruation (1.5%; n=6) |
| Kimmitt 2010 [[40](#_ENREF_40)] | n=2; menstruating (2%), unfamiliar with tampon (2%) |
| Lippman, 2007 [[3](#_ENREF_3)] | Time constraints or prior commitments (9%; n=92) |
| Macmillan 2000 [[22](#_ENREF_22)] | Lack of time (n=17; 34.7%), having period (n=14; 28.6%), unnecessary (n=10; 20.4%) |
| Newman 2003 [[27](#_ENREF_27)] | N=10; uncomfortable (60%), fear or dislike (30%) |
| Richardson 2003 [[16](#_ENREF_16)] | Lack of time (21.4%; n=61), uncomfortable with collection (18.2%; n=52), lack of interest (29.8%; n=85) |
| Serlin 2002 [[25](#_ENREF_25)] | n=34; Lack of time (35%), unwillingness for vaginal swab test (32%), pelvic examination discomfort (12%) |
| Tebb 2004 [[58](#_ENREF_58)] | declined due to lack of time (7%; n=4) |
| Van der Helm 2009 [[35](#_ENREF_35)] | n=66; fear of taking swab incorrectly (66%), Unclear instruction (9%), dislike for self-collection (11%) |
| Wayal 2009 [[36](#_ENREF_36)] | Data not reported in % |

SS: Self-Sample/sampling; CS: Clinician sampling
