# Supplementary material for: Obtaining Self-Samples to Diagnose Curable Sexually Transmitted Infections: A Systematic Review of Patients’ Experiences
Source: PLoS One. 2015 Apr 24;10(4):e0124310. doi: 10.1371/journal.pone.0124310 (PMC4409059; doi:10.1371/journal.pone.0124310)
Supplement: S2 Table — (DOCX) [file pone.0124310.s002.docx]

|  | **Title and Abstract** | **Background rationale** | **Objectives** | **Study Design** | ***Biological sample collection*** | ***Biological sample storage*** | **Setting** | **Participants** | **Variables** | **Data source/ Measurement** | **Bias** | **Study Size** | **Quantitative variables** | **Statistical methods** | **Participants** | | ***Give reason for loss of sample at each stage*** | | **Descriptive data** | | **Outcome data** | | **Main results** | | **Other analyses** | | **Key results** | | **Limitations** | | **Interpretation** | | **Generalisability** | | **Funding** | | ***Ethical committee*** | |
| --- | --- | --- | --- | --- | --- | --- | --- | --- | --- | --- | --- | --- | --- | --- | --- | --- | --- | --- | --- | --- | --- | --- | --- | --- | --- | --- | --- | --- | --- | --- | --- | --- | --- | --- | --- | --- | --- | --- |
| **Markos, 1994 [**[**21**](#_ENREF_21)**]** | √ | x | √ | √ | √ | √ | √ | √ | x | √ | x | √ | x | x | √ | | x | | x | | √ | | x | | x | | √ | | x | | x | | √ | | x | | x | |
| **Macmillan, 2000 [**[**22**](#_ENREF_22)**]** | √ | √ | √ | √ | √ | √ | √ | √ | √ | √ | √ | √ | √ | √ | √ | | √ | | √ | | √ | | √ | | √ | | √ | | √ | | √ | | √ | | √ | | √ | |
| **Stephenson, 2000 [**[**23**](#_ENREF_23)**]** | √ | √ | √ | √ | √ | x | √ | √ | √ | √ | √ | √ | √ | √ | √ | | x | | √ | | √ | | √ | | √ | | √ | | √ | | √ | | √ | | x | | √ | |
| **Fenton, 2001 [**[**56**](#_ENREF_56)**]** | √ | √ | √ | √ | √ | √ | √ | √ | √ | √ | √ | √ | √ | √ | √ | x | | √ | | √ | | √ | | √ | | √ | | √ | | √ | | √ | | √ | | √ | |  |
| **Wiesenfeld, 2001 [**[**47**](#_ENREF_47)**]** | √ | √ | √ | √ | √ | √ | √ | √ | √ | √ | x | √ | √ | √ | √ | | x | | √ | | √ | | √ | | √ | | √ | | x | | √ | | √ | | √ | | √ | |
| **Bloomfield, 2002 [**[**54**](#_ENREF_54)**]** | √ | √ | √ | √ | √ | √ | √ | √ | √ | √ | √ | √ | √ | x | √ | | √ | | √ | | √ | | √ | | √ | | √ | | √ | | √ | | x | | x | | x | |
| **Holland-Hall, 2002 [**[**24**](#_ENREF_24)**]** | √ | √ | √ | √ | √ | √ | √ | √ | √ | √ | √ | √ | √ | √ | √ | | x | | √ | | √ | | √ | | √ | | √ | | √ | | √ | | √ | | √ | | √ | |
| **Serlin, 2002 [**[**25**](#_ENREF_25)**]** | √ | √ | √ | √ | √ | x | x | √ | √ | √ | x | √ | √ | √ | √ | | x | | √ | | √ | | √ | | √ | | √ | | √ | | √ | | √ | | √ | | x | |
| **Bloomfield, 2003 [**[**26**](#_ENREF_26)**]** | √ | √ | √ | √ | √ | √ | √ | √ | √ | √ | x | x | √ | √ | √ | | x | | √ | | √ | | √ | | √ | | √ | | √ | | √ | | √ | | x | | x | |
| **Hsieh, 2003 [**[**48**](#_ENREF_48)**]** | √ | √ | √ | √ | √ | √ | √ | √ | √ | √ | x | √ | √ | √ | √ | | √ | | √ | | √ | | √ | | √ | | √ | | x | | √ | | √ | | √ | | √ | |
| **Newman, 2003 [**[**27**](#_ENREF_27)**]** | √ | √ | √ | √ | √ | x | x | √ | √ | √ | √ | √ | √ | √ | √ | | √ | | √ | | √ | | √ | | √ | | √ | | √ | | √ | | √ | | √ | | √ | |
| **Pimenta, 2003 [**[**28**](#_ENREF_28)**]** | √ | √ | √ | √ | x | x | √ | √ | √ | √ | √ | √ | √ | √ | √ | | √ | | √ | | √ | | √ | | √ | | √ | | √ | | √ | | √ | | √ | | √ | |
| **Richardson, 2003 [**[**16**](#_ENREF_16)**]** | √ | √ | √ | √ | √ | √ | √ | √ | √ | √ | x | √ | √ | √ | √ | | √ | | √ | | √ | | √ | | √ | | √ | | √ | | √ | | √ | | √ | | √ | |
| **Tanksale, 2003 [**[**42**](#_ENREF_42)**]** | √ | √ | √ | √ | √ | √ | √ | √ | √ | √ | X | X | √ | √ | √ | | X | | √ | | √ | | √ | | √ | | √ | | √ | | √ | | √ | | √ | | √ | |
| **Chandeying, 2004 [**[**60**](#_ENREF_60)**]** | √ | √ | √ | √ | √ | √ | √ | √ | √ | √ | x | √ | √ | √ | √ | | x | | √ | | √ | | √ | | √ | | √ | | x | | √ | | x | | √ | | √ | |
| **Tebb, 2004 [**[**58**](#_ENREF_58)**]** | √ | √ | √ | √ | x | x | √ | √ | √ | √ | √ | √ | √ | √ | √ | | √ | | √ | | √ | | √ | | √ | | √ | | √ | | √ | | √ | | √ | | √ | |
|  | **Title and Abstract** | **Background rationale** | **Objectives** | **Study Design** | ***Biological sample collection*** | ***Biological sample storage*** | **Setting** | **Participants** | **Variables** | **Data source/ Measurement** | **Bias** | **Study Size** | **Quantitative variables** | **Statistical methods** | **Participants** | | ***Give reason for loss of sample at each stage*** | | **Descriptive data** | | **Outcome data** | | **Main results** | | **Other analyses** | | **Key results** | | **Limitations** | | **Interpretation** | | **Generalisability** | | **Funding** | | ***Ethical committee*** | |
| **Chernesky, 2005 [**[**29**](#_ENREF_29)**]** | √ | √ | √ | √ | √ | x | √ | √ | √ | √ | √ | √ | √ | √ | √ | | x | | √ | | √ | | √ | | √ | | √ | | x | | √ | | √ | | √ | | √ | |
| **Gotz, 2005 [**[**50**](#_ENREF_50)**]** | √ | √ | √ | √ | √ | x | x | √ | √ | √ | √ | √ | √ | √ | √ | | √ | | √ | | √ | | √ | | √ | | √ | | √ | | √ | | √ | | √ | | x | |
| **Gaydos, 2006 [**[**30**](#_ENREF_30)**]** | √ | √ | √ | √ | √ | √ | √ | √ | √ | √ | √ | √ | √ | √ | √ | | x | | √ | | √ | | √ | | √ | | √ | | √ | | √ | | √ | | √ | | √ | |
| **Hoebe, 2006 [**[**31**](#_ENREF_31)**]** | √ | √ | √ | √ | √ | x | √ | √ | √ | √ | √ | √ | √ | √ | √ | | √ | | √ | | √ | | √ | | √ | | √ | | √ | | √ | | √ | | x | | x | |
| **Van-de-Wijgert, 2006 [**[**32**](#_ENREF_32)**]** | √ | √ | √ | √ | √ | √ | √ | √ | √ | √ | √ | √ | √ | √ | √ | | √ | | √ | | √ | | √ | | √ | | √ | | √ | | √ | | √ | | √ | | √ | |
| **Jones, 2007 [**[**33**](#_ENREF_33)**]** | √ | √ | √ | √ | √ | x | √ | √ | √ | √ | √ | √ | √ | √ | √ | | √ | | √ | | √ | | √ | | √ | | √ | | √ | | √ | | √ | | √ | | √ | |
| **Lippman 2007 [**[**3**](#_ENREF_3)**]** | √ | √ | √ | √ | √ | √ | √ | √ | √ | √ | √ | √ | √ | √ | √ | | √ | | √ | | √ | | √ | | √ | | √ | | √ | | √ | | √ | | √ | | √ | |
| **Papp, 2007 [**[**34**](#_ENREF_34)**]** | √ | √ | √ | √ | √ | √ | √ | √ | √ | √ | √ | √ | √ | √ | √ | | x | | x | | √ | | √ | | √ | | √ | | x | | √ | | x | | x | | √ | |
| **Mahilum Tapay, 2007 [**[**51**](#_ENREF_51)**]** | √ | √ | √ | √ | √ | √ | √ | √ | √ | √ | √ | X | X | X | √ | | √ | | √ | | √ | | √ | | √ | | √ | | √ | | √ | | √ | | √ | | √ | |
| **Berwald, 2009 [**[**55**](#_ENREF_55)**]** | √ | √ | √ | √ | √ | NA | √ | √ | √ | √ | X | X | X | √ | √ | | √ | | √ | | √ | | √ | | √ | | √ | | √ | | √ | | √ | | X | | √ | |
| **van der Helm, 2009 [**[**35**](#_ENREF_35)**]** | √ | √ | √ | √ | √ | x | √ | √ | √ | √ | x | √ | √ | √ | √ | | √ | | √ | | √ | | √ | | √ | | √ | | √ | | √ | | √ | | x | | √ | |
| **Wayal, 2009 [**[**36**](#_ENREF_36)**]**  **Wayal, 2011 [**[**57**](#_ENREF_57)**]** | √ | √ | √ | √ | √ | √ | √ | √ | √ | √ | √ | √ | √ | √ | √ | | √ | | √ | | √ | | √ | | √ | | √ | | √ | | √ | | √ | | √ | | √ | |
| **Brown, 2010 [**[**37**](#_ENREF_37)**]** | √ | √ | √ | √ | x | x | √ | √ | √ | √ | √ | √ | √ | √ | √ | | √ | | √ | | √ | | √ | | √ | | √ | | √ | | √ | | √ | | √ | | √ | |
| **Chai, 2010 [**[**38**](#_ENREF_38)**]** | √ | √ | √ | √ | √ | √ | √ | √ | √ | √ | √ | √ | √ | √ | √ | | √ | | √ | | √ | | √ | | √ | | √ | | √ | | √ | | √ | | √ | | √ | |
| **Dodge, 2010 [**[**17**](#_ENREF_17)**]**  **Rosenberger, 2011 [**[**18**](#_ENREF_18)**]** | √ | √ | √ | √ | x | x | √ | √ | √ | √ | x | √ | √ | x | √ | | x | | √ | | √ | | √ | | √ | | √ | | √ | | √ | | √ | | √ | | √ | |
|  | **Title and Abstract** | **Background rationale** | **Objectives** | **Study Design** | ***Biological sample collection*** | ***Biological sample storage*** | **Setting** | **Participants** | **Variables** | **Data source/ Measurement** | **Bias** | **Study Size** | **Quantitative variables** | **Statistical methods** | **Participants** | | ***Give reason for loss of sample at each stage*** | | **Descriptive data** | | **Outcome data** | | **Main results** | | **Other analyses** | | **Key results** | | **Limitations** | | **Interpretation** | | **Generalisability** | | **Funding** | | ***Ethical committee*** | |
| **Graseck, 2010 [**[**49**](#_ENREF_49)**]** | √ | √ | √ | √ | √ | x | x | √ | √ | √ | √ | √ | √ | √ | √ | | √ | | √ | | √ | | √ | | √ | | √ | | √ | | √ | | √ | | √ | | √ | |
| **Graseck , 2010 [**[**39**](#_ENREF_39)**]** | √ | √ | √ | √ | √ | √ | √ | √ | √ | √ | √ | √ | √ | √ | √ | | √ | | √ | | √ | | √ | | √ | | √ | | √ | | √ | | √ | | √ | | √ | |
| **Kimmitt, 2010 [**[**40**](#_ENREF_40)**]** | √ | √ | √ | √ | √ | √ | √ | √ | X | √ | x | √ | √ | x | √ | | √ | | x | | √ | | √ | | √ | | √ | | √ | | x | | √ | | x | | √ | |
| **Greenland, 2011 [**[**5**](#_ENREF_5)**]** | √ | √ | √ | √ | √ | X | √ | √ | √ | √ | X | X | √ | √ | √ | | X | | √ | | √ | | √ | | √ | | √ | | √ | | √ | | X | | √ | | X | |
| **Huppert, 2011 [**[**19**](#_ENREF_19)**]** | √ | √ | √ | √ | √ | NA | √ | √ | √ | √ | X | X | √ | √ | √ | | √ | | √ | | √ | | √ | | √ | | √ | | √ | | √ | | √ | | √ | | √ | |
| **Freeman, 2011 [**[**46**](#_ENREF_46)**]** | √ | √ | √ | √ | √ | √ | √ | √ | √ | √ | X | X | √ | √ | √ | | X | | √ | | √ | | √ | | X | | √ | | √ | | √ | | √ | | X | | √ | |
| **Huppert, 2012 [**[**20**](#_ENREF_20)**]** | √ | √ | √ | √ | √ | X | √ | √ | √ | √ | X | X | √ | √ | √ | | √ | | √ | | √ | | √ | | √ | | √ | | √ | | √ | | √ | | √ | | X | |
| **Reagan, 2012 [**[**41**](#_ENREF_41)**]** | √ | √ | √ | √ | √ | X | √ | √ | √ | √ | √ | √ | √ | √ | √ | | √ | | √ | | √ | | √ | | √ | | √ | | √ | | √ | | X | | √ | | √ | |
| **Kwan, 2012 [**[**53**](#_ENREF_53)**]** | √ | √ | √ | √ | √ | X | √ | √ | √ | √ | X | X | √ | √ | √ | | √ | | √ | | √ | | √ | | √ | | √ | | √ | | √ | | √ | | X | | X | |
| **Kohil, 2013 [**[**59**](#_ENREF_59)**]** | √ | √ | √ | √ | √ | X | √ | √ | √ | X | X | X | X | X | √ | | √ | | √ | | √ | | √ | | √ | | √ | | √ | | √ | | X | | X | | √ | |
| **Roth, 2013 [**[**43**](#_ENREF_43)**]** | √ | √ | √ | √ | √ | X | √ | √ | √ | √ | X | √ | √ | √ | √ | | √ | | √ | | √ | | √ | | √ | | √ | | √ | | √ | | √ | | X | | √ | |
| **Gudka, 2013 [**[**44**](#_ENREF_44)**]** | √ | √ | √ | √ | √ | X | √ | √ | √ | √ | X | X | √ | √ | √ | | √ | | √ | | √ | | √ | | √ | | √ | | √ | | √ | | X | | √ | | √ | |
| **Gaydos, 2013 [**[**30**](#_ENREF_30)**]** | √ | √ | √ | √ | √ | X | √ | √ | √ | √ | √ | X | √ | √ | √ | | √ | | √ | | √ | | √ | | √ | | √ | | √ | | √ | | X | | √ | | √ | |
| **Fielder, 2013 [**[**52**](#_ENREF_52)**]** | √ | √ | √ | √ | √ | X | √ | √ | √ | √ | X | X | √ | X | √ | | √ | | X | | √ | | √ | | √ | | √ | | √ | | √ | | √ | | √ | | √ | |

Note: √ reported, X not reported
